# KIF2A regulates the spindle assembly and the metaphase I-anaphase I transition in mouse oocyte

**DOI:** 10.1038/srep39337

**Published:** 2016-12-19

**Authors:** Ming-Huang Chen, Yu Liu, Ya-Long Wang, Rui Liu, Bai-Hui Xu, Fei Zhang, Fei-Ping Li, Lin Xu, Yan-Hong Lin, Shu-Wen He, Bao-Qiong Liao, Xian-Pei Fu, Xiao-Xue Wang, Xiang-Jun Yang, Hai-Long Wang

**Affiliations:** 1 Organ Transplantation Institute, Medical College, Xiamen University, Xiamen 361000, Fujian, China; 2 Department of Gynaecology and Obstetrics, Zhongshan Hospital, Xiamen University, Xiamen 361000, Fujian, China; 3 Fujian Key Laboratory of Organ and Tissue Regeneration, Xiamen 361000, Fujian, China; 4 Department of Gynaecology and Obstetrics, Zhongxin Hospital, Qingdao 266000, Shandong, China; 5 Biological College, Southwest Forestry University, Kunming 650000, Yunnan, China; 6 Department of Gynaecology and Obstetrics, The First Clinical Medical College, Fujian Medical University, Fuzhou 350000, Fujian, China; 7 Department of Gynaecology and Obstetrics, Dongfang Hospital, Xiamen University, Fuzhou 350000, Fujian, China

## Abstract

KIF2A, a member of the kinesin-13 family, has been reported to play a role in spindle assembly in mitosis. However, its function in mammalian meiosis remains unknown. In this research, we examined the expression, localization and function of KIF2A during mouse oocyte meiosis. KIF2A was expressed in some key stages in mouse oocyte meiosis. Immunofluorescent staining showed that KIF2A distributed in the germinal vesicle at the germinal vesicle stage and as the spindle assembling after meiosis resumption, KIF2A gradually accumulated to the entire spindle. The treatment of oocytes with taxol and nocodazole demonstrated that KIF2A was co-localized with α-tubulin. Depletion of KIF2A by specific short interfering (si) RNA injection resulted in abnormal spindle assembly, failure of spindle migration, misaligned chromosomes and asymmetric cell division. Meanwhile, SKA1 expression level was decreased and the TACC3 localization was disrupted. Moreover, depletion of KIF2A disrupted the actin cap formation, arrested oocytes at metaphase I with spindle assembly checkpoint protein BubR1 activated and finally reduced the rate of the first polar body extrusion. Our data indicate that KIF2A regulates the spindle assembly, asymmetric cytokinesis and the metaphase I-anaphase I transition in mouse oocyte.

In mammalian oocytes, meiosis is a crucial event for the success of keeping genomic stability. Prior to fertilization, oocytes undergo two consecutive divisions without an intervening replicative process^1^. In contrast to the mitosis, the division in mammalian oocytes maturation is highly asymmetric giving rise to a large and highly polarized oocyte and a tiny polar body. This process plays a critical role in female meiosis to ensure that most of the maternal components can be retained within the oocyte for the early embryo development^2^. Failure of asymmetric division is a remarkable characteristic of low-quality oocytes^3^.

In meiosis, spindle assembly, spindle positioning and cortical polarity compose the three basic requirements that finally promote the extrusion of an asymmetric polar body. The spindle assembly is directed by Microtubule organizing centers (MTOCs) which consist of a pair of centrioles with the pericentriolar material (PCM) surrounded in most animal cells. During mammalian oocyte maturation, however, the MTOCs do not have centrioles^4^,^5^,^6^,^7^. In mouse oocyte, under the special direction, microtubules accumulate around the condensed chromosomes to form a bipolar spindle near the cell center after germinal vesicle breakdown (GVBD). Then the meiotic spindle migrates to the periphery of the oocyte in a microfilament-dependent but not a microtubule-dependent way^8^,^9^. As the spindle migrating to the cortex, the formation of an actin cap and a cortical granule-free domain (CGFD) was induced over the spindle^8^,^10^. Thus the proteins involved in the three essential events above are likely to play a certain role in mammalian oocytes maturation, sequentially affecting the fate of mouse oocyte.

KIF2A is a member of the kinesin-13 family that belongs to kinesin superfamily proteins (KIFs) which are ubiquitous in all eukaryotic organisms. KIFs are a class of microtubule-dependent molecular motor proteins which share a highly conserved motor domain and were proved to have motion characteristics and adenosine triphosphatase (ATPase) activity^11^. Kinesins play a key role in many kinds of cellular processes including the intracellular transport of vesicles and organelles, cytokinesis, signal transduction and cell morphogenesis^12^,^13^,^14^. Referring to the functions in the mitosis, kinesins have various roles involving centrosome separation, chromosome attachment to the microtubules, chromosome congression, sister chromatid segregation and maintenance of bipolar spindle^15^,^16^,^17^. The kinesin-13 proteins, one branch of KIFs, are the important regulators in microtubule dynamics during mitosis^18^. This branch of the kinesin superfamily proteins can promote microtubule depolymerization through disassembly tubulin subunits from the polymer end^18^. In human genome, the kinesin-13 family has four distinct genes encoding members including KIF2A, KIF2B, MCAK/KIF2C and KIF24. It is found that KIF2A is required for the formation of bipolar spindle through a functional relationship with MCAK during mitosis^19^. In human U2OS cells, KIF2A plays an essential role in the poleward microtubule flux and maintaining the spindle length by depolymerizing the minus ends of microtubules at the spindle pole^20^,^21^. Furthermore, the antagonistic regulation of KIF2A by Plk1 and Aurora A confers spatial differential stability to microtubules which is crucial for the mitotic spindle assembly and function^22^.

Although KIF2A has been widely investigated in mitosis, the role in meiosis still remains unclear. In this study we explore the expression, localization and the role of KIF2A during mouse oocyte maturation. Our results demonstrate that KIF2A regulates the spindle assembly, asymmetric cytokinesis and metaphase I-anaphase I transition in mouse oocyte.

## Results

### Expression and subcellular localization of KIF2A during meiotic maturation in mouse oocytes

**Figure 1:**
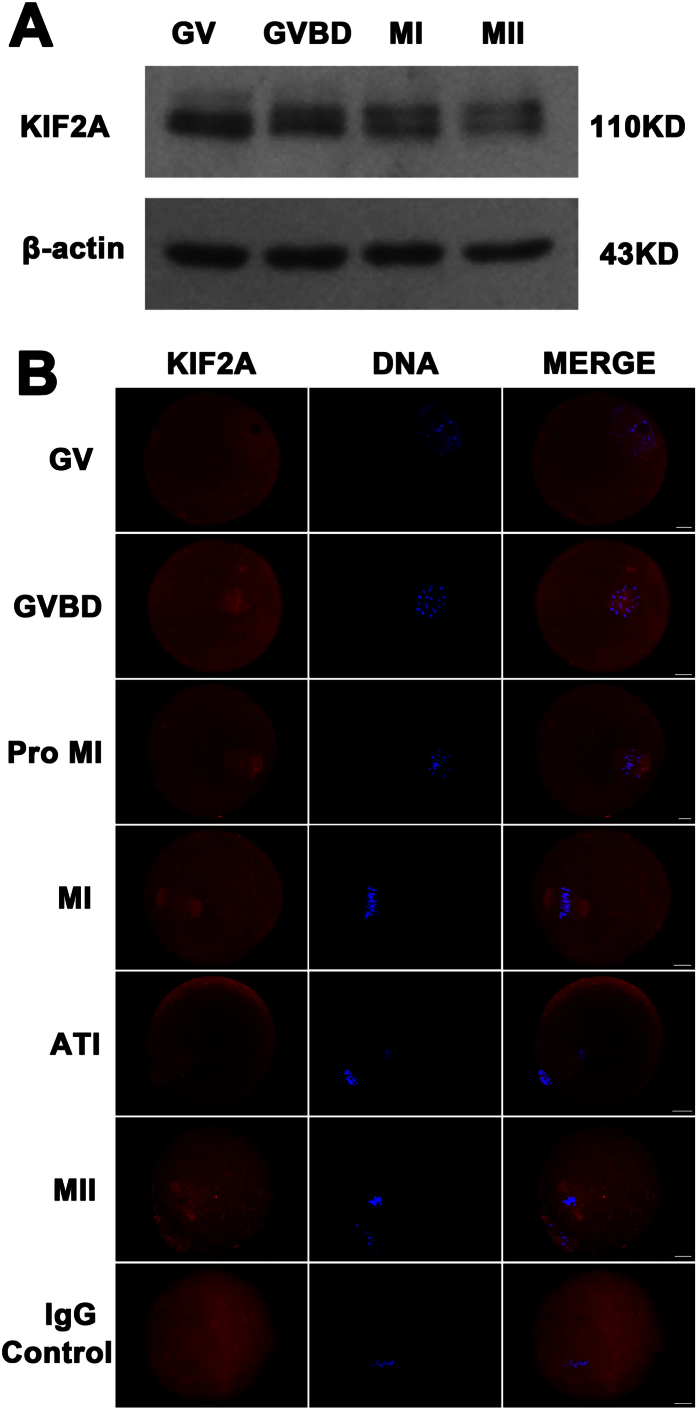
Expression and subcelluar localization of KIF2A in mouse oocyte meiosis. (**A**) Representative images of KIF2A protein level at GV, GVBD, MI and MII stages. KIF2A was expressed stably at GV, GVBD and MI stages, but decreased slightly at MII stage. Full-length blots are presented in Supplementary Figure 1. (**B**) Oocytes at various stages were stained with an anti-KIF2A antibody (red) and each sample was co-stained with DAPI to detect DNA (blue). KIF2A was localized to the germinal vesicle at GV stage, and then was localized to the entire spindle. Scale bar = 20 μm.

Oocytes were cultured for 0 h, 2 h, 6.5 h, 8.5 h, 10 h and 12 h, corresponding to most oocytes had reached germinal vesicle (GV), germinal vesicle breakdown (GVBD), pro-metaphase I (Pro-MI), metaphase I (MI), anaphase-telophase I (ATI) and metaphase II (MII), respectively. Western blotting was used to detect the KIF2A expression. As shown in Fig. 1A, KIF2A expression was maintained in an approximate level from GV stage to MI stage while a slight decrease was detected at MII stage. Then KIF2A immunofluorescent staining was used to investigate the subcellular localization of KIF2A at different stages of meiotic maturation in mouse oocytes. As shown in Fig. 1B, KIF2A signal was detected in the germinal vesicle at the GV stage. After GVBD, KIF2A gradually aggregated to the periphery of chromosomes. Subsequently, the signal of KIF2A was detected stably on the entire spindle at the Pre-MI, MI, ATI and MII stages. In the IgG control group, no obvious signals can be detected.

**Figure 2:**
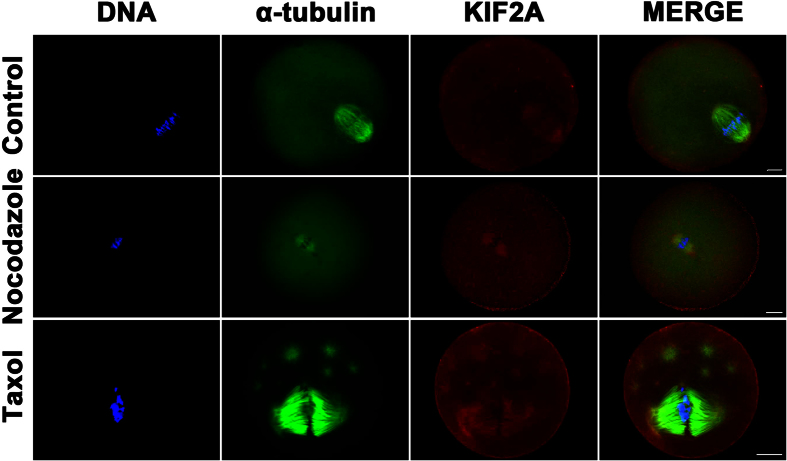
Localization of KIF2A in mouse oocytes treated with spindle-perturbing agents. MI oocytes were incubated in M2 medium with 20 μg/ml nocodazole for 10 min and 50 μM taxol for 45 min, respectively. Oocytes were treated with the same amount of DMSO and the same time in the control. The oocytes were collected for immunostaining. KIF2A was stably co-localized with α-tubulin. Green α-tubulin, red KIF2A, blue DNA. Scale bar = 20 μm.

To further investigate the correlation between KIF2A localization and spindle microtubule dynamics, spindle-perturbing agents were employed to treat oocytes that were at the MI stage. As shown in Fig. 2, nocodazole, a microtubule-depolymerizing agent, was first used to treat oocytes. After treatment, the microtubules were disassembled and the spindle was turned into a small one. Nevertheless, KIF2A still localized to the entire spindle. Taxol was then used to treat oocytes at the MI stage. Taxol can promote microtubule assembly and stabilize polymerized microtubules. After taxol treatment, excessive polymerization of the microtubules was induced, resulting in multiple asters and obviously enlarged spindles in mouse oocytes. In our case, KIF2A signals were observed on the cytoplasmic asters and the enlarged spindles.

### Depletion of KIF2A caused abnormal positioning and assembly of spindle and misaligned chromosomes in mouse oocytes

**Figure 3:**
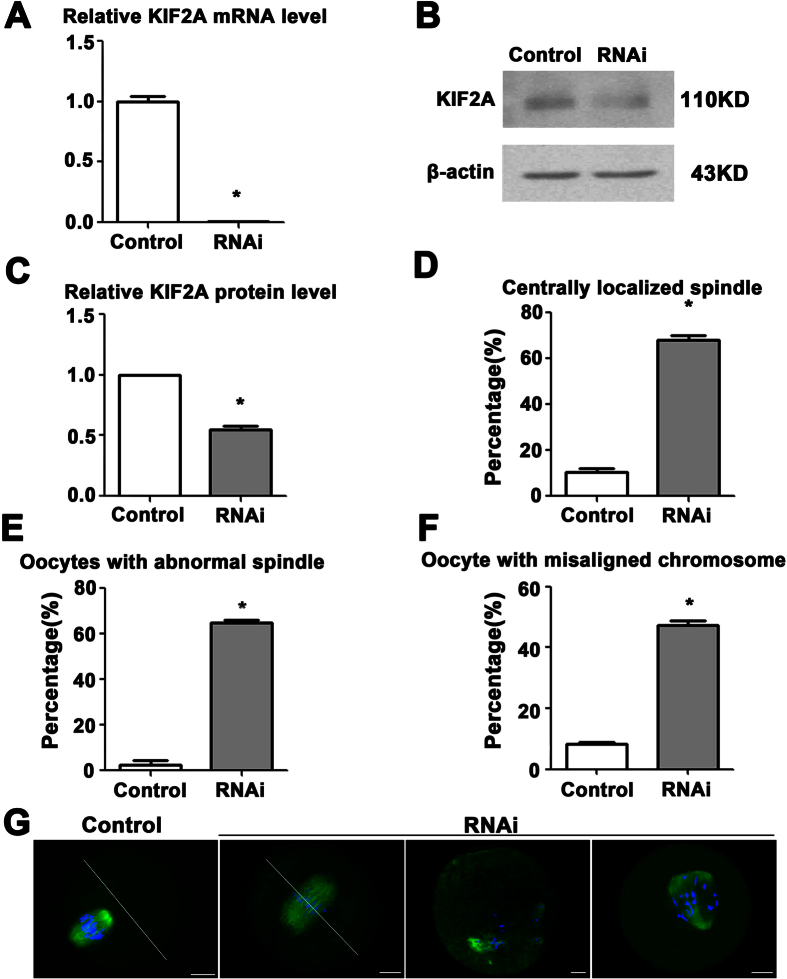
Depletion of KIF2A caused abnormal spindle and misaligned chromosomes in mouse oocytes. (**A**) The level of KIF2A mRNA in the KIF2A siRNA-injected groups is significantly lower than the control. (**B**) Representative images of KIF2A protein level in the KIF2A and control siRNA-injected groups. Full-length blots are presented in Supplementary Figure 2. (**C**) Relative level of KIF2A protein in the KIF2A siRNA-injected groups is significantly lower than the control. (**D**) Percentage of oocytes with centrally localized spindles in the KIF2A siRNA-injected groups was higher than the control. (**E**) Percentage of oocytes with malformed spindles in the KIF2A siRNA-injected groups was higher than the control. (**F**) Percentage of oocytes with misaligned chromosomes in the KIF2A siRNA-injected groups was higher than the control. Data are presented as the mean ± SEM of three independent experiments, ^*^P < 0.05. (**G**) Representative images of chromosomes and spindle morphology in the KIF2A and control siRNA-injected groups. Green α-tubulin, blue DNA. Scale bar = 20 μm.

To explore the function of KIF2A in mouse oocyte meiosis, we employed siRNA injection to knock down the expression of KIF2A. Oocytes from the KIF2A and control siRNA-injected groups were harvested for the real-time qPCR analysis in order to detect the relative level of KIF2A mRNA. The relative level of KIF2A mRNA in the KIF2A siRNA-injected groups was significantly decreased as compared to the control (0.96 ± 0.01% vs. 100.20 ± 4.29%, n = 150; P < 0.05) (Fig. 3A). And then western blotting was employed to determine the protein level of KIF2A. The protein level of KIF2A was depressed successfully in the KIF2A siRNA-injected groups (Fig. 3B). Grey level analysis indicated that the relative level of KIF2A protein in the KIF2A siRNA-injected groups was significantly reduced as compared to the control (54.96 ± 2.63% vs. 100.00 ± 0.00%, n = 600; P < 0.05) (Fig. 3C).

After 8.5 h of culture, the oocytes were collected for immunostaining of α-tubulin and DNA in order to examine the spindle positioning and assembly in the KIF2A and control siRNA-injected groups. As shown in Fig. 3D,G, most oocytes exhibited normal peripheral spindle positioning in the control, while spindles in a large proportion of oocytes still stayed near the oocyte center in the KIF2A siRNA-injected groups (67.88 ± 2.17%, n = 156 vs. control 10.64 ± 1.24%, n = 150; P < 0.05). Moreover, the assembly of spindle was impaired seriously in the KIF2A siRNA-injected groups (Fig. 3G). Spindles that exhibited multipole, monopole, no pole or abnormal shape were identified as abnormal spindles. The rate of oocytes with abnormal spindle in the KIF2A siRNA-injected groups was significantly higher than the control (64.94 ± 1.20%, n = 160 vs. control 2.64 ± 1.72%, n = 151; P < 0.05) (Fig. 3E). Meanwhile, oocytes with misaligned chromosomes were notably higher in the KIF2A siRNA-injected groups (47.39 ± 1.33%, n = 116 vs. control 8.38 ± 0.64%, n = 131; P < 0.05) (Fig. 3F).

### Depletion of KIF2A decreased SKA1 expression level and disrupted TACC3 localization

**Figure 4:**
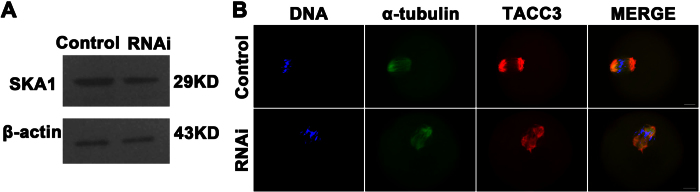
Depletion of KIF2A decreased SKA1 expression level and disrupted TACC3 localization in mouse oocytes. (**A**) Representative images of SKA1 protein level in the KIF2A and control siRNA-injected groups. Full-length blots are presented in Supplementary Figure 3. (**B**) Representative images of TACC3 localization in the KIF2A and control siRNA-injected groups. TACC3 was localized to the spindle poles in the control groups. However, it’s disrupted in the KIF2A siRNA-injected groups. Red TACC3, green α-tubulin, blue DNA. Scale bar = 20 μm.

To explore which mechanism KIF2A works through, MI oocytes were collected for western blotting of SKA1 and immunostaining of TACC3. As shown in Fig. 4A, SKA1 expression level was decreased slightly in the KIF2A siRNA-injected groups. As shown in Fig. 4B, TACC3 localization was disrupted severely in the KIF2A siRNA-injected groups while TACC3 was localized to the spindle poles in the control groups.

### Depletion of KIF2A decreased PB1 extrusion and caused failure of asymmetric cytokinesis

**Figure 5:**
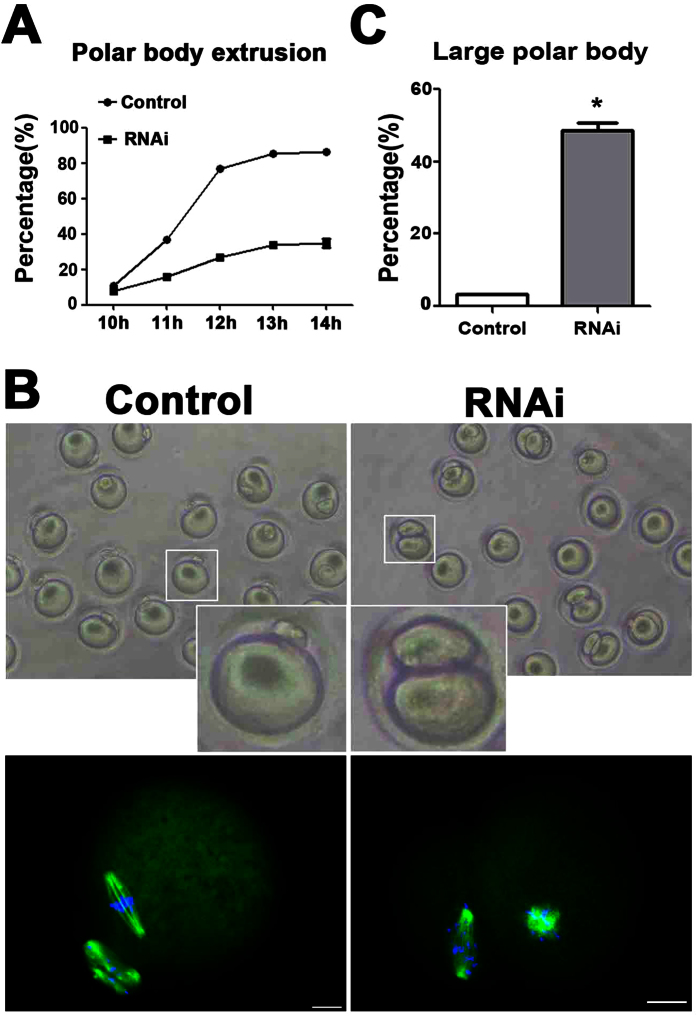
Depletion of KIF2A affected first polar body extrusion and asymmetric division in mouse oocytes. (**A**) Percentage of first polar body (PB1) extrusion in the KIF2A siRNA-injected groups was lower than the control at various stages. (**B**) Representative images of PB1 in the KIF2A and control siRNA-injected groups. Green α-tubulin, blue DNA. Scale bar = 20 μm. (**C**) Percentage of large PB1 in the KIF2A siRNA-injected groups was higher than the control. Data are presented as the mean  ±  SEM of three independent experiments, ^*^P < 0.05.

To further explore the function of KIF2A in mouse oocyte meiosis, oocytes were cultured in fresh M2 medium for 10 h, 11 h, 12 h, 13 h and 14 h after siRNA injection. As shown in Fig. 5A, the rate of the PB1 extrusion in the KIF2A siRNA-injected groups was decreased significantly as compared to the control. We then dissected the oocytes that extruded the first polar body in both the KIF2A and control siRNA-injected groups. We found that a large proportion of oocytes failed to accomplish asymmetric division in the KIF2A siRNA-injected groups (Fig. 5B). As shown in Fig. 5C, a significantly larger proportion of oocytes in the KIF2A siRNA-injected groups emitted large PB1. (48.74 ± 1.93%, n = 147 vs. control 3.34 ± 0.73%, n = 199; P < 0.05).

### Depletion of KIF2A disrupted actin cap formation in mouse oocytes

**Figure 6:**
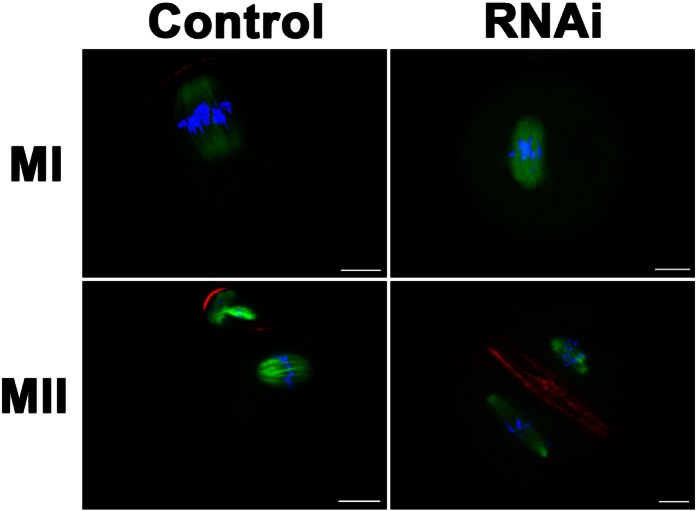
Depletion of KIF2A affected the formation of actin cap in mouse oocytes. Representative images of actin cap in the KIF2A and control siRNA-injected groups. It can be clearly observed in the control groups. However, we can’t detect the actin cap in the KIF2A siRNA-injected groups. Green α-tubulin, red actin, blue DNA. Scale bar = 20 μm.

The disruption of oocyte polarity may be responsible for the defects in spindle migration and asymmetric cytokinesis. Hence, we examined the formation of actin cap, a remarkable characteristic of mouse oocyte polarization. Oocytes were cultured for 8.5 h and 12 h, corresponding to MI and MII, respectively. As shown in Fig. 6, the spindle in the control had already migrated to the cortex and an actin cap formed over the spindle at the later MI stage, but in the KIF2A siRNA-injected groups, the spindle remained near the oocyte center and no actin cap can be detected. In the KIF2A siRNA-injected groups, the MII oocytes formed a 2 half-size structure with no typical actin cap. In the control groups, on the contrary, oocytes exhibited normal MII morphology.

### Depletion of KIF2A caused MI arrest in mouse oocytes

**Figure 7:**
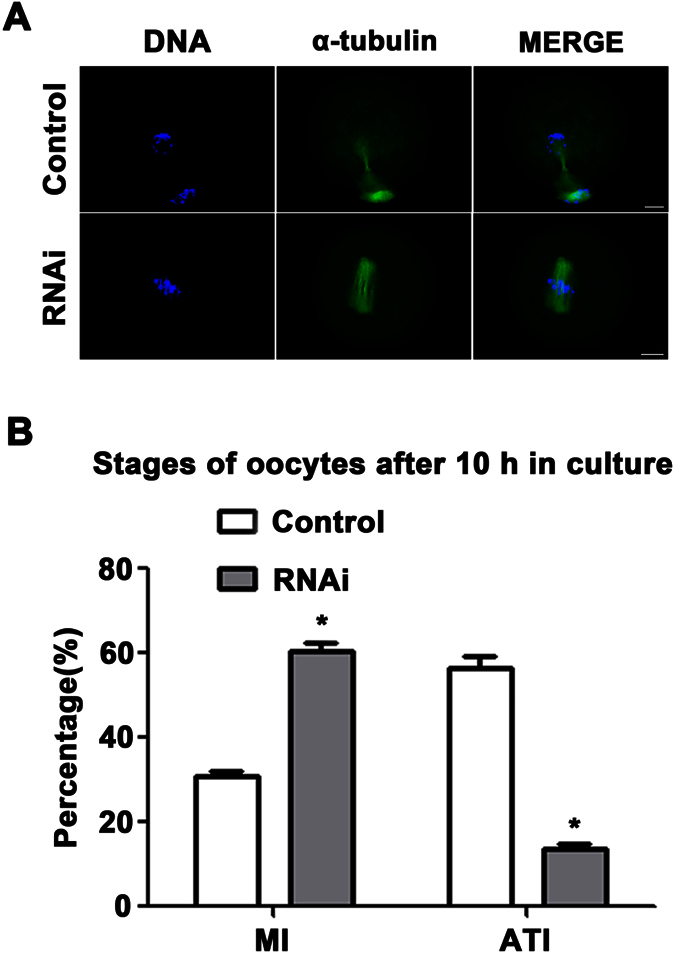
Depletion of KIF2A arrested oocytes at MI stage. (**A**) Oocytes in the KIF2A siRNA-injected groups were arrested at MI stage after 10 h of culture. The oocytes had progressed to ATI stage in the control group. Green α-tubulin, blue DNA. Scale bar = 20 μm. (**B**) Percentage of oocytes at MI and ATI stages in the KIF2A and control siRNA-injected groups. In the KIF2A siRNA-injected groups, more oocytes were arrested at MI stage and fewer oocytes had progressed to ATI stage. Data are presented as the mean ± SEM of three independent experiments. MI: RNAi vs. Control ^*^P < 0.05; ATI: RNAi vs. Control ^*^P < 0.05.

To further explore in which link KIF2A functions concretely in mouse oocytes maturation cycle, oocytes were cultured for 10 h after microinjection. Then oocytes were collected for immunostaining to assess oocytes stages. The rate of oocytes arrested at MI stage was significantly higher in the KIF2A siRNA-injected groups (60.66 ± 1.64%, n = 183 vs. control 31.04 ± 0.83%, n = 222; P < 0.05) (Fig. 7A,B), while a larger proportion of oocytes have already progressed into anaphase-telophase I in the control (56.40 ± 3.01%, n = 222 vs. 13.66 ± 1.09%, n = 183; P < 0.05) (Fig. 7A,B).

### Depletion of KIF2A activated the spindle assembly checkpoint (SAC) protein in mouse oocytes

**Figure 8:**
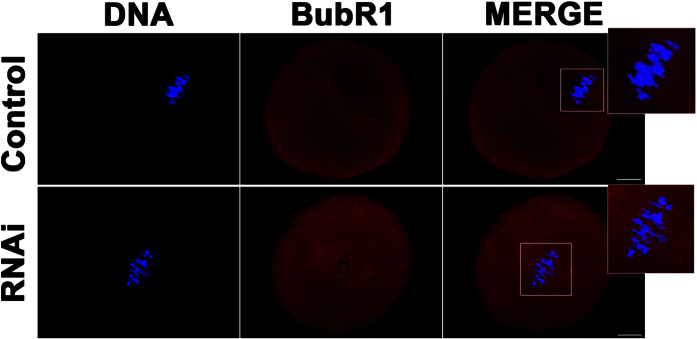
Depletion of KIF2A activated the SAC protein in mouse oocytes. Representative images of BubR1 localization in the KIF2A and control siRNA-injected groups. BubR1 was localized to the kinetochores in the KIF2A siRNA-injected groups. It can’t be observed in the control groups. Red BubR1, blue DNA. Scale bar = 20 μm.

To investigate whether the result above is due to the activation of SAC, oocytes were cultured for 8.5 h, and then stained to detect the signal of BubR1. As shown in Fig. 8, evident signals of BubR1 were observed in the KIF2A siRNA-injected groups, indicating the activation of the spindle assembly checkpoint (SAC) protein, while no BubR1 signals were observed in the control groups.

## Discussion

In this study, we explored the expression, localization patterns and potential roles of KIF2A for the first time during the mouse oocyte meiotic maturation. Our data have shown that perturbation of KIF2A function causes spindle assembly and positioning defects, misaligned chromosomes, cell progression disorder and failure of asymmetric cell division.

As we know, the role of one protein can be generally inferred through its localization. Previous studies have shown that in human CFPAC-1cells, KIF2A not only is localized to the centrosomes in interphase, but also is localized to spindle microtubules being concentrated at poles. And it is localized to the spindle midzone and midbody in addition to the spindle poles in anaphase and telophase^19^. This study demonstrated that KIF2A was expressed in some key stages in mouse oocyte maturation. Our results, however, showed that KIF2A was localized to the entire meiotic spindle after GVBD. The localization pattern of KIF2A is similar to some other proteins that have been reported to be involved in spindle assembly such as ERK3 and Septin1^23^,^24^, indicating that KIF2A might play a role in the microtubule organization and spindle assembly. In addition, as nocodazole and taxol treatment inducing abnormal spindle assembly, KIF2A was found to be co-localize with α-tubulin in the spindle and asters, also supporting it.

We successfully knocked down the KIF2A expression level by siRNA microinjection to explore the effect of KIF2A on spindle assembly in mouse oocyte meiosis. A large proportion of oocytes with spindle defects and misaligned chromosomes were found in the KIF2A siRNA-injected groups. It has been reported that KIF2A regulates spindle assembly in mitosis through a functional relationship with MCAK which is the best characterized member of the kinesin-13 family^19^. However, the mechanism in meiosis still needs further exploration. The proper assembly of bipolar spindle is the result of the cooperation of motor and nonmotor proteins such as TACC, NuMA and TPX2^25^,^26^,^27^. TACC3, a member of TACC, has been reported to play an important role in meiosis^28^. And in other studies, SKA1 exhibits the similar localization with KIF2A in mouse oocyte and SKA1 regulates spindle dynamics with KIF2A in mitosis^29^,^30^. In our study, depletion of KIF2A also disrupted the TACC3 localization and decreased SKA1 expression level. And in mitosis, Nuf2 RNAi can rescue the spindle defects in the KIF2A-depleted cells^31^. Thus KIF2A is likely to function through the interaction with Nuf2, TACC, NuMA, SKA1 or TPX2 during oocyte meiosis.

Interestingly, a large proportion of oocytes exhibited centrally localized spindle and extruded large polar body in the KIF2A-depleted groups. And the formation of actin cap was disrupted. Spindle migration, cortical spindle positioning, cortical reorganization and oocyte polarization determine the oocyte asymmetric cytokinesis^3^. Our results have demonstrated that most oocytes in KIF2A-depleted group failed to achieve asymmetric cell division. The spindle migration pattern in *Drosophila melanogaster and C. elegans* is dependent on the microtubule-severing enzyme MEI-1 and microtubules^32^,^33^. However, it is dependent on c-mos and F-actin but not microtubules in mouse^34^. It has been reported that the spindle migration is dependent on a dynamic actin meshwork around the meiotic spindle in many species^35^,^36^. And our previous publication has reported that WHAMM localizes around the meiotic spindle and regulates spindle migration and asymmetric cytokinesis in mouse oocytes^37^. SKA1, JMY and Formin-2 exhibit similar localization with KIF2A and participate in spindle migration^29^,^38^,^39^. Arp2/3 plays a key role in this process but it does not localize to the spindle^40^,^41^. Thus KIF2A might regulate the spindle migration through interacting with the proteins above.

Furthermore, knockdown of KIF2A in mouse oocytes significantly reduced the proportion of oocytes that extruded PB1 and led to MI arrest and activation of SAC, indicating that it inflicted serious disorder on the cell cycle progression. Considering that depletion of KIF2A disrupted the spindle assembly, improper attachments of microtubules to kinetochores might occur, resulting in the activation of SAC followed by failure of the MI-AI transition^42^,^43^,^44^. There’s another possibility that KIF2A plays a role in the SAC proteins transport from kinetochores.

In conclusion, we investigated the potential roles of KIF2A during mouse oocyte maturation. Our data demonstrated that KIF2A is required for the spindle assembly which is essential for the chromosome congression and the metaphase I-anaphase I transition in mouse oocytes. In addition, it can affect the spindle migration and the formation of actin cap to regulate asymmetric cytokinesis.

## Materials and Methods

### Ethics statement

All experiments including the care and use of mice were conducted in strict accordance with the approval in the Guide for the Care and Use of Laboratory Animals of the National Institutes of Health. The protocol in this study was approved by Animal Studies Committee of Xiamen University, China (approval ID: XMUMC 2011-10-08). ICR mice were raised in a constant-temperature room with a regular diet. Cervical dislocation was performed to minimize the suffering of mice and the only procedure implemented on the dead mice was to get ovaries and harvest oocytes from the ovaries.

### Antibodies and reagents

A rabbit polyclonal anti-KIF2A antibody was purchased from Novus Biologicals (Littleton, CO, USA). Normal rabbit IgG was purchased from Santa Cruz Biotechnology (Dallas, CA, USA). A rabbit polyclonal anti-SKA1 antibody was purchased from Abcam (Cambridge, MA, USA). A rabbit monoclonal anti-TACC3 antibody was purchased from Abcam (Cambridge, MA, USA). Mouse monoclonal FITC-conjugated anti-α-tubulin antibody, TRITC-conjugated phalloidin, nocodazole and taxol were purchased from Sigma-Aldrich (St Louis, MO, USA). Sheep polyclonal anti-BubR1 antibody was purchased from Abcam (Cambridge, MA, USA). Alexa Fluor 546-conjugated donkey anti-sheep and donkey anti-rabbit antibodies were obtained from Invitrogen (Carlsbad, CA, USA). M2 medium was purchased from Sigma-Aldrich (St Louis, MO, USA). Milrinone was obtained from Cayman Chemical Co. (Ann Arbor, MI, USA). Mounting medium containing DAPI was obtained from Vector Laboratories (Burlingame, CA, USA). Horseradish peroxidase (HRP)-conjugated mouse anti-rabbit IgG and horseradish peroxidase (HRP)-conjugated rabbit anti-mouse IgG were obtained from Zhong Shan Jin Qiao Co. (Beijing, P. R. China). A mouse monoclonal anti-β-actin antibody was obtained from Abcam (Cambridge, MA, USA). Enhanced chemiluminescence (ECL) detection substrate was obtained from Xiamen Lulong Biotech Co. (Xiamen, P. R. China).

### Oocyte harvest and culture

Germinal vesicle (GV)-intact oocytes were harvested from the ovaries of female ICR mice which are 6 to 8 weeks old in M2 medium supplemented with or without 2.5 μM milrinone. The oocytes were then cultured in fresh M2 medium under mineral oil in a 5% CO_2_ incubator, maintaining at 37 °C in humidified air. Oocytes were collected for further analysis at different stages during culture.

### Nocodazole treatment

Stock 10 mg/ml nocodazole in dimethyl sulfoxide (DMSO) was diluted in M2 medium to generate a final concentration of 20 μg/ml. MI stage oocytes were then cultured in this medium for 10 min. The oocytes in the control group were cultured in the M2 medium with the same amount of DMSO. Oocytes were collected for immunofluorescence after treatment.

### Taxol treatment

Stock taxol (5 mM in DMSO) was diluted to generate a final concentration of 50 μM. MI stage oocytes were then cultured in this medium for 45 min. The oocytes in the control group were cultured in the M2 medium with the same amount of DMSO. Oocytes were collected for immunofluorescence after treatment.

### KIF2A siRNA microinjection

Fully grown GV stage oocytes were microinjected with aliquots of 5–10 pL of 50 μM KIF2A siRNA (5′-GGA UGU UGA UGC UAC AAA UTT-3′, 5′-AUU UGU AGC AUC AAC AUC CTT-3′) (GenePharma, Shanghai, P. R. China) or negative control siRNA (5′-UUC UCC GAA CGU GUC ACG UTT-3′, 5′-ACG UGA CAC GUU CGG AGA ATT-3′) (GenePharma, Shanghai, P. R. China). The process was performed with a Narishige MM0-202N hydraulic three-dimensional micromanipulator (Narishige Inc., Sea Cliff, NY, USA) under the Nikon Diaphot ECLIPSE TE300 inverted microscope (Nikon UK Ltd., Kingston upon Thames, Surrey, UK). The oocytes were cultured for 24 h in M2 medium with 2.5 μM milrinone after microinjection. After three 2-min washes in fresh M2 medium, the oocytes were then cultured in fresh M2 medium under mineral oil in a 5% CO_2_ incubator, maintaining at 37 °C in humidified air. Oocytes were collected at different stages for further analysis.

### Real-time quantitative polymerase chain reaction (PCR) analysis

Real-time quantitative PCR and the ΔΔC_T_ method normalized by β-actin were employed to measure the KIF2A gene expression. Each sample contained 50 oocytes, and total RNA was extracted by a Dynabead mRNA DIRECT kit (Life Technologies AS, Oslo, Norway). A cDNA synthesis kit (Toyobo, Osaka, Japan) was used to generate the first strand cDNA with Oligo (dT) 12–18 nucleotide primers (Takara Bio Inc., Tokyo, Japan). The following primers were used to amplify the KIF2A and β-actin cDNA fragments:

KIF2A forward, 5′-GACCTGGCTGGGAACGAAAG-3′

KIF2A reverse, 5′- TTTGTTTCTACCTAAGGCTCGGATG-3′

β-actin forward, 5′-CATCCGTAAAGACCTCTATGCCAAC-3′

β-actin reverse, 5′- ATGGAGCCACCGATCCACA-3′

The SYBR Green real-time PCR Master Mix kits (Life Technologies, Carlsbad, CA, USA) was utilized with a Step One real-time PCR system (Applied Biosystems, Foster City, CA, USA). The following conditions were used to perform the process: 50 °C for 2 min and 95 °C for 2 min; followed by 40 cycles of 95 °C for 15 s and 60 °C for 1 min.

### Western blotting

Each sample containing 200 oocytes was collected in sodium dodecyl sulfate (SDS) loading buffer and heated for 5 minutes at 100 °C. After separated by SDS-PAGE, the proteins were electrically transferred to polyvinylidene fluoride (PVDF) membranes. The membranes were blocked in TBS containing 0.1% Tween and 5% skimmed milk for 1 hr at room temperature. Then the membranes were incubated overnight at 4 °C with 1:3000 rabbit anti-KIF2A antibody, 1:500 rabbit anti-SKA1 antibody or 1:10000 mouse anti-β-actin antibody. After three 10-min washes in TBS containing 0.1% Tween, the membranes were incubated for 1 hr at room temperature with 1:10000 horseradish peroxidase (HRP)-conjugated mouse anti-rabbit IgG and 1:10000 horseradish peroxidase (HRP)-conjugated rabbit anti-mouse IgG, respectively. Then three 10-min washes were performed on the membranes. Finally, the enhanced chemiluminescence (ECL) detection substrate was used to process the membranes.

### Immunofluorescence and confocal microscopy

Our previous publications have described the detailed methods^37^,^45^. For single immunostaining of KIF2A, normal rabbit IgG, BubR1 or α-tubulin, 4% paraformaldehyde in phosphate-buffered saline (PBS) was used to fix oocytes for 30 min at room temperature. After being permeabilized in PBS supplemented with 0.5% Triton X-100 for 30 min at room temperature, oocytes were blocked in PBS supplemented with 1% bovine serum albumen (BSA) for 1 h and incubated for 1 h at room temperature with 1:200 mouse anti-α-tubulin-FITC antibody or overnight at 4 °C with 1:25 diluted rabbit anti-KIF2A antibody, 1:50 normal rabbit IgG, 1:25 sheep polyclonal anti-BubR1 antibody. After three 5-min washes in PBS containing 0.1% Tween 20 and 0.01% Triton X-100, the oocytes were incubated for 1 h at room temperature with 1:500 donkey Anti-Rabbit IgG antibodies conjugated with Alexa Fluor 546 (to stain KIF2A) or 1:250 donkey anti-sheep IgG antibodies conjugated with Alexa Fluor 546 (to stain BubR1) (this step in immunostaining of α-tubulin was omitted).

For double immunostaining of KIF2A and α-tubulin, after KIF2A immunostaining, the oocytes were washed in washing buffer (PBS containing 0.1% Tween 20 and 0.01% Triton X-100) for three times and then blocked again in PBS supplemented with 1% BSA for 1 h at room temperature, followed by immunostaining with 1:200 mouse FITC-conjugated anti-α-tubulin antibody for 1 h at room temperature.

For double immunostaining of TACC3 and α-tubulin, after TACC3 (1:50) immunostaining, the oocytes were washed in washing buffer (PBS containing 0.1% Tween 20 and 0.01% Triton X-100) for three times and then blocked again in PBS supplemented with 1% BSA for 1 h at room temperature, followed by immunostaining with 1:200 mouse FITC-conjugated anti-α-tubulin antibody for 1 h at room temperature.

For double immunostaining of actin and α-tubulin, the oocytes were placed in phalloidin conjugated with TRITC (10 μg/ml) for 30 min. After three 5-min washes, they were blocked again in PBS supplemented with 1% BSA for 1 h at room temperature, followed by immunostaining with 1:200 mouse FITC-conjugated anti-α-tubulin antibody for 1 h at room temperature.

After three 5-min washes in washing buffer as above, the oocytes were then mounted onto glass slides using mounting medium with DAPI for immunostaining of DNA. A FV1000 confocal laser-scanning microscope (Olympus, Tokyo, Japan) was finally used to examine the oocytes.

### Statistical analysis

At least three replications were implemented for each experiment. Data (mean ± standard error of the mean, SEM) were analyzed by ANOVA using Graph-Pad Prism software (v. 5; La Jolla, CA, USA) followed by Student’s *t* tests. Difference at P < 0.05 was regarded to be statistically significant. The number of oocytes was labeled in parentheses as (n).

## Additional Information

**How to cite this article**: Chen, M.-H. *et al*. KIF2A regulates the spindle assembly and the metaphase I-anaphase I transition in mouse oocyte. *Sci. Rep.* **6**, 39337; doi: 10.1038/srep39337 (2016).

**Publisher's note:** Springer Nature remains neutral with regard to jurisdictional claims in published maps and institutional affiliations.

## Supplementary Material

Supplementary Information
