# Supplementary material for: KIF2A regulates the spindle assembly and the metaphase I-anaphase I transition in mouse oocyte
Source: Sci Rep. 2016 Dec 19;6:39337. doi: 10.1038/srep39337 (PMC5171862; doi:10.1038/srep39337)
Supplement: Supplementary Information [file srep39337-s1.pdf]

KIF2A regulates the spindle assembly and the metaphase I-anaphase I transition in mouse oocyte

Ming-Huang Chen[1,2,3], Yu Liu[1,3], Ya-Long Wang[1,3], Rui Liu[2,3,7], Bai-Hui Xu[1,3], Fei Zhang[2,3], Fei-Ping Li[3,4], Lin Xu[1,3], Yan-Hong Lin[3,5], Shu-Wen He[1,3], Bao-Qiong Liao[3,6], Xian-Pei Fu[1,3], Xiao-Xue Wang[2], Xiang-Jun Yang[2*] & Hai-Long Wang[1,3*]

[1] Organ Transplantation Institute, Medical College, Xiamen University, Xiamen 361000, Fujian, China

[2] Department of Gynaecology and Obstetrics, Zhongshan Hospital, Xiamen University, Xiamen 361000, Fujian, China

[3]Fujian Key Laboratory of Organ and Tissue Regeneration, Xiamen 361000, Fujian, China

[4] Biological College, Southwest Forestry University, Kunming 650000, Yunnan, China

[5] Department of Gynaecology and Obstetrics, The First Clinical Medical College, Fujian Medical University, Fuzhou 350000, Fujian, China

[6] Department of Gynaecology and Obstetrics, Dongfang Hospital, Xiamen University, Fuzhou 350000, Fujian, China

[7] Department of Gynaecology and Obstetrics, Zhongxin Hospital, Qingdao 266000, Shandong, China

*****Corresponding author. Tel.: +86-592-2880568; Fax: +86-592-2188421.

E-mail address: hailongwang@xmu.edu.cn (H.-L. Wang).

*****Alternate corresponding author. Tel.: +86-592-2187157; Fax: +86-592-2187157.

E-mail address: xingyun207@126.com (X.-J. Yang).

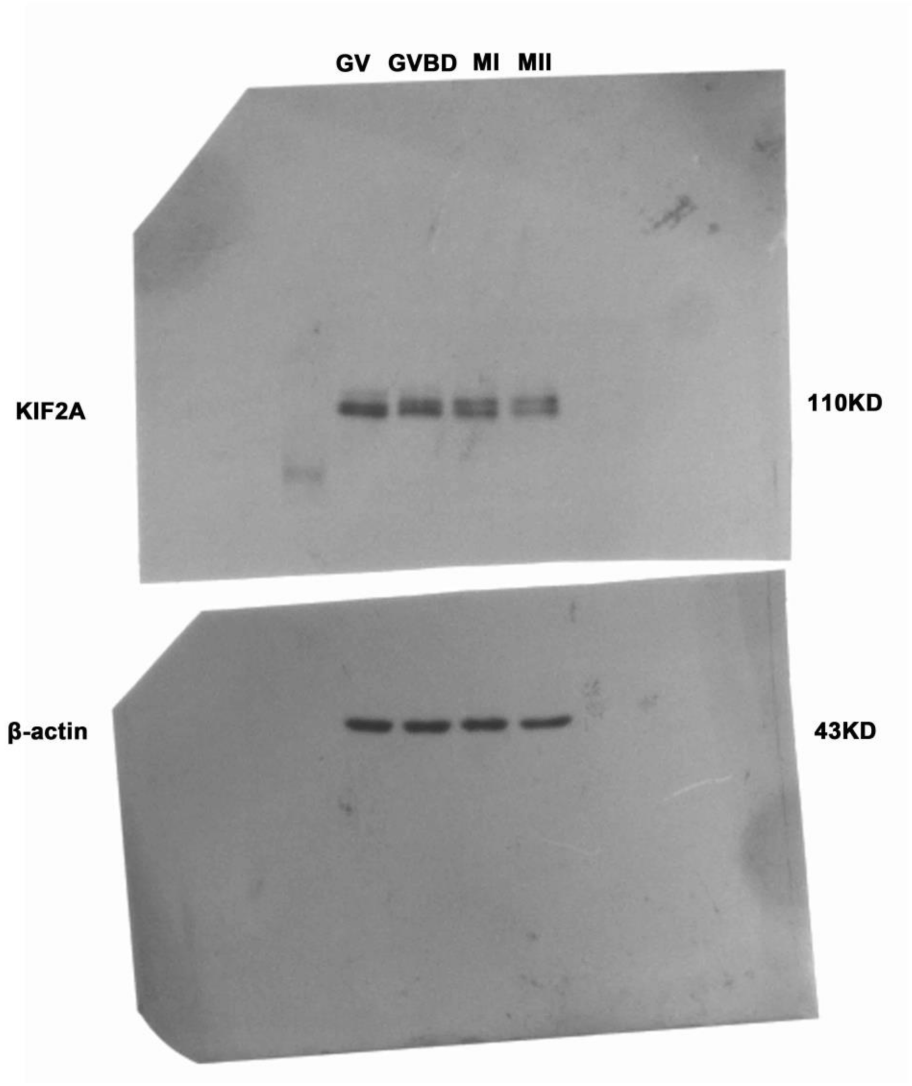

Supplementary Figure 1. Expression of KIF2A at GV, GVBD, MI and MII stages. Each sample contained 200 oocytes. The molecular size of KIF2A and β-actin are 110KD and 43KD, respectively.

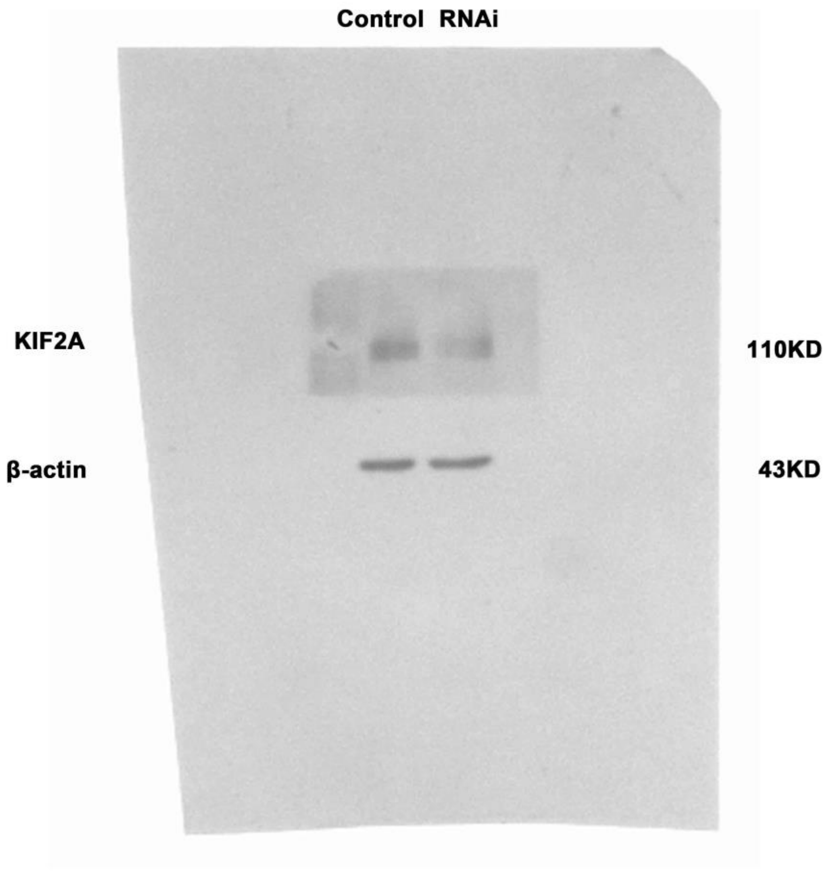

Supplementary Figure2. Expression of KIF2A in the control and KIF2A siRNA-injected group. Oocytes were cultured to MI stage and then were collected for western blotting. Each sample contained 200 oocytes. The molecular size of KIF2A and β-actin are 110KD and 43KD, respectively.

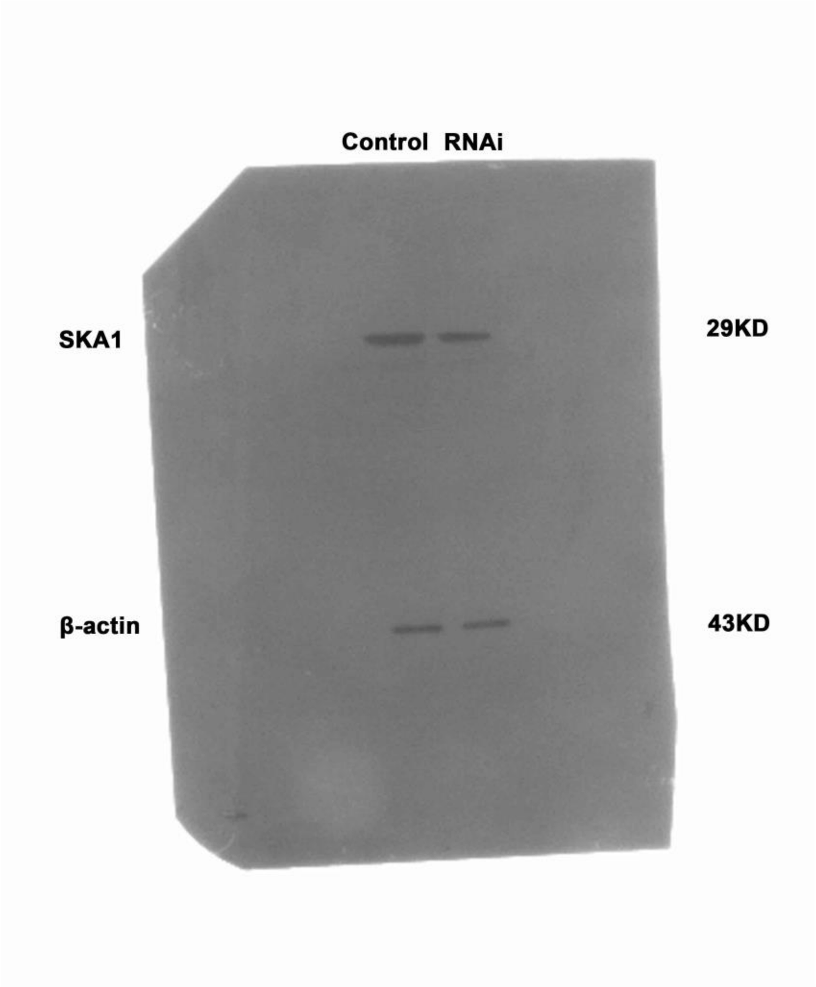

Supplementary Figure 3. Expression of SKA1 in the control and KIF2A siRNA-injected group. Oocytes were cultured to MI stage and then were collected for western blotting. Each sample contained 200 oocytes. The molecular size of SKA1 and β-actin are 29KD and 43KD, respectively.
